# The input of organic fertilizer can improve soil physicochemical properties and increase cotton yield in southern Xinjiang

**DOI:** 10.3389/fpls.2024.1520272

**Published:** 2025-01-10

**Authors:** Yupeng Zhao, Qingyong Bian, Zhiduo Dong, Xiaojuan Rao, Zhiguo Wang, Yanbo Fu, Bolang Chen

**Affiliations:** ^1^ Institute of Soil Fertilizer and Agricultural Water Conservation, Xinjiang Academy of Agricultural Sciences, Urumqi, Xinjiang, China; ^2^ College of Resources and Environment, Xinjiang Agricultural University, Urumqi, China; ^3^ Baicheng Agricultural Experimental Station/National Soil Quality Aksu Observation Experimental Station, Xinjiang Academy of Agricultural Sciences, Aksu/National Soil Quality Observation and Experimental Station, Aksu, Xinjiang, China; ^4^ Biological Science and Technology College of Xinjiang Agricultural Vocational and Technical University, Urumqi, Xinjiang, China

**Keywords:** soil nutrients, cotton, soil physical and chemical properties, soil microorganisms, southern Xinjiang

## Abstract

In this study, the improvement effect of different organic substances on compacted cohesive soil in southern Xinjiang was discussed, with emphasis on the influence of different organic substances on soil chemical properties and microorganisms, so as to determine the best carbon source input and provide theoretical support for the rational utilization of organic materials in southern Xinjiang. Field experiments were conducted to evaluate the effects of farm fertilizer, biochar, commercial organic fertilizer, microbial fertilizer and mineral potassium humate on physical and chemical properties of viscous soil, agronomic properties and yield of cotton, with three gradients for each organic fertilizer. The results showed that: (1) all organic fertilizers improved soil structure, among which farm fertilizer significantly reduced soil bulk density and salinity, increased soil organic matter, total nitrogen and available nutrients, and thus increased cotton height, stem diameter and yield. The optimal application amount was 36000 kg/hm². (2) The application of different organic matter increased the contents of organic matter, total nitrogen, hydrolyzed nitrogen, available phosphorus and available potassium in 0-40 cm soil layer, increased the number of bacteria, fungi and actinomyces, and reduced soil salinity; (3) Structural equation model (SEM) was used to investigate the effect mechanism of organic matter input on soil microbial quantity, soil physicochemical properties and cotton yield. The model further confirmed the mechanism: the input of organic matter mainly regulates the number of microorganisms and the richness of microbial species, thereby improving the physical and chemical properties of soil and thereby increasing the cotton yield. The addition of 5 kinds of organic materials can promote the growth and yield of cotton. The comprehensive evaluation shows that the improvement effect is best when the fertilizer dosage is 150% of the recommended amount. In summary, as an effective soil amendment, farm manure can not only alleviate soil compaction, but also significantly improve the growth potential of cotton, which is in line with the goal of sustainable agricultural development.

## 1 Introduction

Soil is a basic resource for food production and is critical to environmental quality and the health of plants, animals and humans. Soil compaction is an obvious manifestation of soil degradation, which can be caused by a variety of factors such as heavy machinery operations, improper farming practices, soil acidification, and salinization caused by excessive use of fertilizers. These problems can lead to soil structural deterioration and compaction (Głąb, 2014; Han, 2015; Figueiredo et al., 2017), which in turn increases soil hardness and reduces its ability to store, retain and transport water. This compaction leads to decreased soil productivity, weakened water and nutrient storage capacity, and ultimately requires more fertilizer inputs, increasing production costs. In addition, soil compaction interferes with the carbon and nitrogen cycles, inhibits the mineralization of organic matter (De Neve and Hofman, 2000), increases the concentration of carbon dioxide in the soil (Conlin, 2000), and reduces microbial activity (Hamza and Anderson, 2005). Compaction may also cause soil erosion, resulting in nutrient loss and increasing environmental pollution (Du et al., 2022).The southern region of Xinjiang is located in an extremely arid climate, where evaporation is greater than precipitation, which makes the deep saline water in farmland gradually rise to the soil surface with evaporation or plant transpiration, resulting in secondary salinization of farmland soil. Saline-alkali soil contains a large number of sodium ions, chloride ions, these ions have a strong ability to disperse soil particles, resulting in a high degree of dispersion of soil particles, will destroy the soil aggregate structure, make the soil permeability and permeability become poor, the soil wet sticky or dry hard. On the whole, soil compaction leads to a serious decline in cultivated land quality and constraints on comprehensive agricultural production capacity, which directly threatens China’s food security and poses challenges to sustainable agricultural development (Figueiredo et al., 2017). Therefore, improving soil quality has become an urgent problem in the current land use and management.

The use of organic materials can reduce the amount of fertilizer used, improve soil fertility, thereby increasing crop yields and improving the ecological environment. Wen et al (Wen et al., 2015). showed that compared with the single application of chemical fertilizer, single application of organic fertilizer or combined application of organic and inorganic fertilizer could significantly improve the level of soil nutrients. The soil organic matter content increased by 95% to 136%, the total nitrogen content increased by 69% to 137%, the available phosphorus content increased by about five times, the available potassium content increased by 81% to 103%, and the pH value decreased by 0.15 to 0.47. The study of Qiu et al (Qiu et al., 2019). showed that different types of organic fertilizer application could effectively improve soil fertility, and the yield of maize in the current season was increased by 48.60% and the economic benefit was increased by 33.75% compared with conventional fertilization. In addition, the application of organic fertilizer not only improves the soil nutrients and microbial quantity, but also enhances the water retention ability (Unger, 1978; Haynes and Naidu, 1998), activates the activity of soil enzymes, and promotes the decomposition and release of organic matter (Stemmer et al., 1998).

Long-term use of organic materials can further improve the nitrogen use efficiency of crops, increase yield, reduce the loss of nitrogen and carbon, and improve the ecological environment. Understanding the characteristics of different types of organic materials and rational use are of great significance for improving soil fertility and crop yield. However, previous studies on the application of organic materials in corn fields (Cheng et al., 2021; Cui et al., 2022; Li et al., 2022) mainly focused on livestock manure, crop straw and biogas residue green manure. In this study, five kinds of organic materials, including organic fertilizer, biochar, microbial fertilizer, commercial organic fertilizer and fulvic acid, were applied in different gradients to investigate their effects on the physical and chemical properties of clay soil, crop agronomic traits and yield.

## 2 Materials and methods

### 2.1 Profile of The test area

Field experiments were conducted from April to October 2023 in Kuqa City, Aksu Prefecture, Xinjiang (41°45′N, 83°25′E). Aksu Prefecture is located in the central part of southern Xinjiang, characterized by high terrain in the north and lower elevations in the south. The northern region is marked by numerous peaks, while the southern area features the vast Taklamakan Desert. The central area consists of piedmont gravel fans, alluvial plains, and interspersed gobi and oasis landscapes.

The region exhibits typical characteristics of a warm temperate continental arid climate, with low precipitation and significant seasonal variability. The annual precipitation ranges from 53.2 to 120.6 mm, and there is an abundance of groundwater resources, with water tables ranging from 2 to 7 meters deep. The area experiences many sunny days and long daylight hours, with annual sunlight hours between 2,670 and 3,022 hours, and total solar radiation of 5,340 to 6,220 MJ/m², providing ample light and heat resources. The temperature exhibits a large annual and diurnal range, with an average annual temperature of 7.9 to 13.7°C, and a frost-free period lasting 168 to 225 days. The experimental area utilized drip irrigation, with no winter or spring irrigation.

In March 2023, the physical and chemical properties of the test soil were measured, revealing an organic matter content of 11.7 g/kg, soil moisture content of 12%, bulk density of 1.50 g/cm³, hydrolyzable nitrogen content of 31.2 mg/kg, available phosphorus content of 12.4 mg/kg, available potassium content of 223.6 mg/kg, and soluble salt content of 11.8 g/kg.The number of soil bacteria was 1.05×108CFU g^-1^, the number of fungi was 1.0567×108CFU g^-1^, and the number of actinomyces was 2.4633×108CFU g^-1^.

### 2.2 Test materials

The experimental materials included five different organic materials: farmyard manure (N, decomposed cattle manure), biochar (T), microbial fertilizer (J), commercial organic fertilizer (S), and mineral potassium humate (H). The cotton seeds used for testing were the ‘Xinluzao 41’ variety, purchased from Xinjiang Tianyu Seed Industry Co., Ltd (Kuqa City, Xinjiang). The organic matter and main nutrient contents of each organic material are detailed in **Table 1**.

**Table 1:** Basic physical and chemical properties of the test materials.

Materials for testing	Organic matter (%)	Nitrogen (%)	Phosphorus (%)	Potassium (%)
Farm manure	77.2	2.27	1.1	2.54
Biochar	65	2.9	2.4	3.5
Biological bacterial fertilizer	60	3.1	1.9	3.7
Commodity organic fertilizer	50	1.7	2.4	2.8
Source of potassium fulvic acid	70	3.3	4.5	2.7

### 2.3 Test design

The experimental site was located in Duntuoktan Town, Kuqa City, Aksu Prefecture. The experiment included five treatments and one control, specifically farmyard manure (N), biochar (T), microbial fertilizer (J), commercial organic fertilizer (S), mineral potassium humate (H), and a control (CK). The application rates for each treatment are shown in **Table 2**, where the 100% recommended rate was determined based on surveys conducted with local farmers.

**Table 2:** The amount of fertilizer applied in each treatment.

Treatment	Fertilizer application rate		
	50% of the recommended amount	100% of the recommended amount	150% of the recommended amount
Farm manure (Kg HM^-2^)	12000	24000	36000
Biochar (Kg HM^-2^)	15000	30000	45000
Biological bacterial fertilizer (Kg HM^-2^)	3750	7500	11250
Commodity organic fertilizer (Kg HM^-2^)	1500	3000	4500
Source of potassium fulvic acid (Kg HM^-2^)	375	750	1125

Each experimental plot measured 5 m × 6 m, with a total area of 30 m². Each treatment included three application rates: 50% recommended rate (1), 100% recommended rate (2), and 150% recommended rate (3), with three replications for each rate, See **Table 2** for specific dosage. Cotton was sown on April 10, 2023, and the organic materials were applied once on April 5, 2023. Following this, the management of the experimental area was carried out by farmers based on their usual farming practices to ensure a high germination rate of the cotton.

### 2.4 Indicators and methods of project determination

#### 2.4.1 Soil samples

On October 5, 2023, during the cotton harvesting period, soil samples of 0-20 and 20-40cm were randomly taken with soil drill at five points (Wei et al., 2024), and soil pH was adopted by glass electrode method (water and soil mass ratio 1:5). The ratio of soil and deionized water was 1:5, and the mixture was shaken for 0.5 h. After filtration, it was determined by conductivity meter(DDSJ-308F measuring tester). The quality method of soil soluble salt was adopted. The soil alkali-hydrolytic nitrogen content was used by alkali-diffusion method. The content of available phosphorus was extracted by 0.5mol·L-1NaHCO3 colorimetric method. The content of available potassium was determined by ammonium acetate extraction and flame photometer. The content of organic matter was determined by potassium dichromate method. Soil total nitrogen was measured by semi-automatic nitrogen analyzer(DNN-04A) (Bao, 2000).

Equation 1.


(1) pd =M/V

Where, pd is the bulk density of a soil layer (g/cm3); M is the mass (g); V is the unit volume (cm3).

Equation 2 (Liu, 1982).


(2) $$\begin{array}{c} Soil\;porosity\;=\left(1\;-\;bulk\;weight/specific\;gravity\right)\\ \times 100\%;The\;specific\;gravity\;of\;soil\;is\;approximately\;2.65\;g/cm^{3} \end{array}$$

#### 2.4.2 Soil microbial sample

On October 5, 2023, during the early cotton harvest, soil samples were collected from five random points at a depth of 0–40 cm using a soil auger. The quantities of bacteria, fungi, and actinomycetes were determined using the dilution plate method. Soil dilution series (dilution levels 10^-^¹ to 10^-6^) were prepared and inoculated onto solid culture media plates, which were then incubated at 28–30°C for 3–6 days. The microbial counts (CFU) in three adjacent dilution levels of the soil solution were recorded, and the number of microorganisms per gram of dry soil was calculated (expressed in CFU/g). The bacterial medium used was beef extract peptone agar; the fungal medium used was Bengal rose agar; and the actinomycete medium used was an improved Gause No. 1 medium (Li et al., 2022).

#### 2.4.3 Plant samples

During the seedling stage of cotton, three plants were randomly sampled from each treatment using a five-point method. Each part of the samples was brought back to the lab to measure plant height and stem diameter, as well as fresh weights of aboveground and underground parts. The samples were then subjected to a killing treatment at 105°C for 30 minutes, followed by drying at 70°C until constant weight was achieved, and the dry weights of the aboveground and underground parts were recorded (Sun et al., 2021).

#### 2.4.4 Yield and yield components

Cotton yield was measured by randomly selecting three representative sampling points within each plot, using either a diagonal method or a five-point star method. To avoid edge effects, a measurement area of 1 m × 2.3 m was selected in the center of each plot to record the number of plants and bolls, allowing for the calculation of harvest density (plants/hm²). From each plot, 15 fully opened cotton bolls were harvested to determine the weight of single-plant bolls and calculate cotton yield (Qu et al., 2021).

#### 2.4.5 Calculation method

Seed cotton yield (kg hm-2) = harvest density (kg hm-2) × average boll number per plant (plant-1) × boll weight per plant (g)/1000 × correction coefficient (90%).

### 2.5 Data processing

Data analysis was conducted using Microsoft Excel 2020, while SPSS 25.0 was used for one-way ANOVA, correlation analysis, and significance testing. Graphs were created using Origin 2018.

## 3 Results

### 3.1 Impact of different treatments on soil physical and chemical properties

#### 3.1.1 Impact of different treatments on soil bulk density and porosity

**Figure 1:**
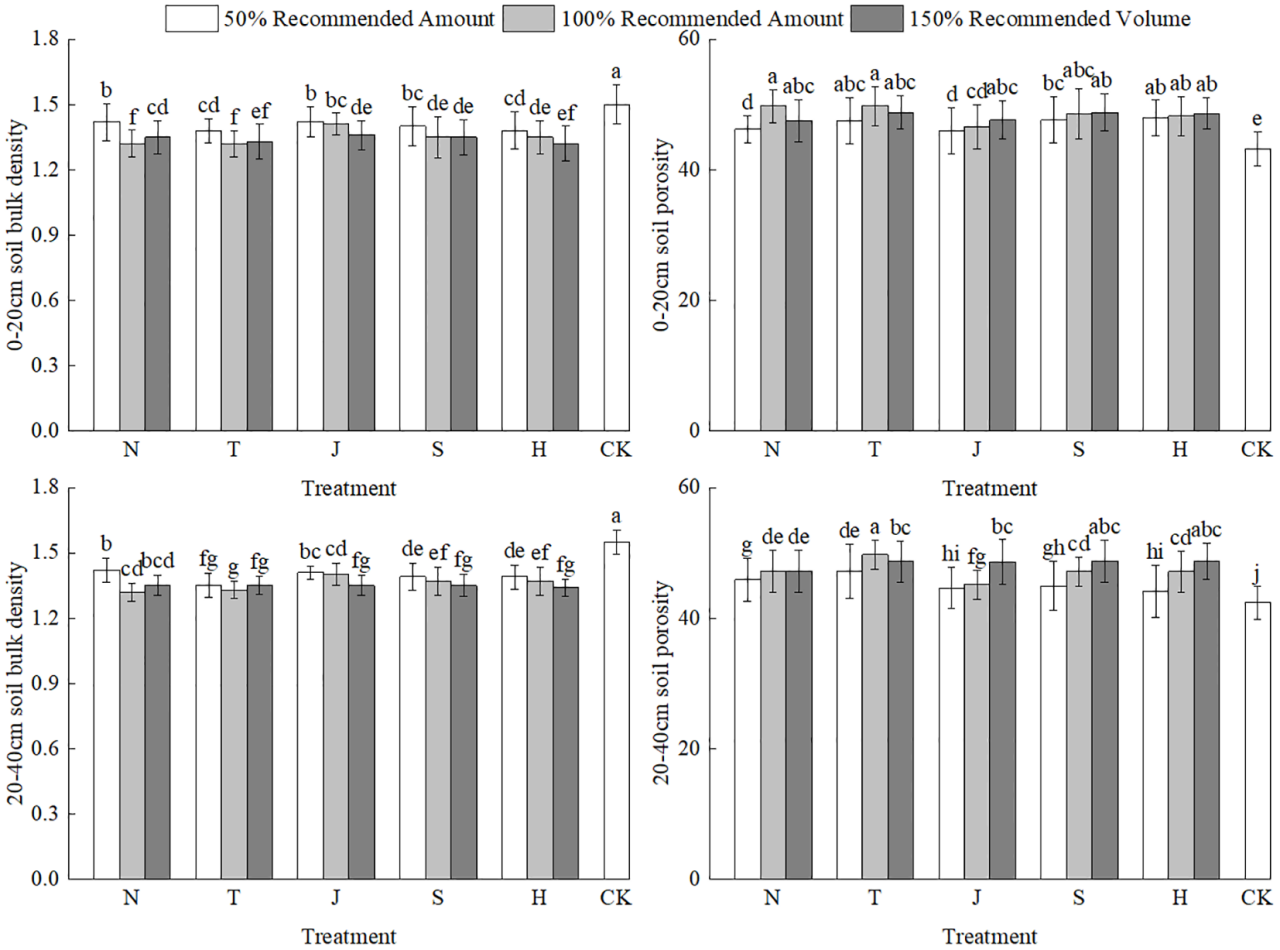
Effects of different organic materials on soil bulk density and porosity. Different lowercase letters indicate significant differences at the 0.05 probability level (P < 0.05), determined by one-way analysis of variance (ANOVA) and Duncan’s *post hoc* test for significance. The vertical bar chart represents the mean ± standard deviation (SD) calculated from three repetitions.N: farm fertilizer, T: biochar, J: biological bacterial fertilizer, S: commercial organic fertilizer, H: mineral source potassium fulvic acid.

From (**Figure 1**), it can be observed that with the application of different organic materials, all treatments significantly reduced the bulk density of the 0–40 cm soil layer. This indicates that farmyard manure, biochar, microbial fertilizers, commercial organic fertilizers, and humic acid effectively improved soil structure. Notably, the N2 and T2 treatments in the N and T groups showed particularly pronounced improvements in bulk density. However, with increasing application rates, the N3 and T3 treatments exhibited a rise in soil bulk density, while the bulk density in the J, S, and H groups consistently decreased with higher application rates. Specifically, the T2 treatment achieved the best improvement, with bulk density reductions of 12.78% for the 0–20 cm layer and 10.71% for the 20–40 cm layer compared to the CK treatment.

The application of different organic materials also significantly affected the soil porosity of the 0–40 cm layer. In the 0–20 cm layer, the N2 and T2 treatments resulted in the most notable increases in soil porosity, with increases of 14.97% and 15.22%, respectively, compared to the CK treatment. In the J, S, and H groups, the J3, S3, and H3 treatments showed significant differences from the other gradient treatments, increasing porosity by 17.34%, 18.02%, and 18.14%, respectively, compared to the CK treatment. This indicates that the addition of organic materials can effectively enhance the total porosity of the soil, thereby improving its aeration capacity.

#### 3.1.2 Effects of different treatments on soil nutrients and salinity

**Figure 2:**
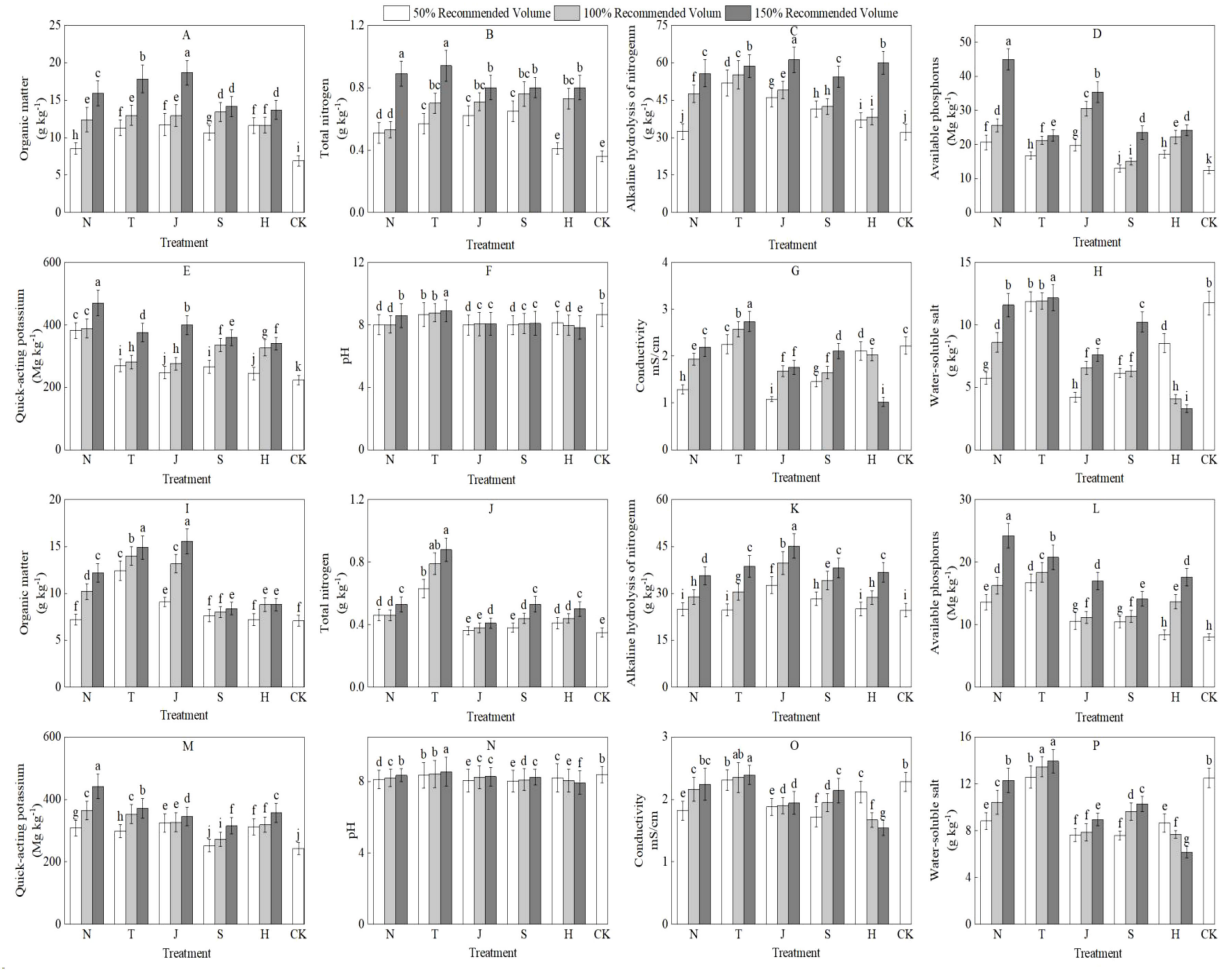
Effects of different organic materials on soil nutrients and salinity. Different lowercase letters indicate significant differences at the 0.05 probability level (P < 0.05), determined by one-way analysis of variance (ANOVA) and Duncan’s *post hoc* test for significance. The vertical bar chart represents the mean ± standard deviation (SD) calculated from three repetitions.N: farm fertilizer, T: biochar, J: biological bacterial fertilizer, S: commercial organic fertilizer, H: mineral source potassium fulvic acid,0-20: **(A–H)**. 20-40: **(I–P)**.

From (**Figure 2**), it can be observed that the application of organic materials led to increases in the content of organic matter, total nitrogen, hydrolyzable nitrogen, available phosphorus, and available potassium in the 0-20 cm soil layer. Compared to the control treatment (CK), there were significant differences in the effects of various treatments on different nutrients. Specifically, the J3 treatment had the most pronounced effect on enhancing organic matter content, while the N1 treatment showed the smallest increase. The T3 treatment exhibited the greatest increase in total nitrogen, whereas the H1 treatment had the smallest increase. For hydrolyzable nitrogen, the J3 treatment again demonstrated the largest increase, while the N1 treatment had the smallest. Regarding available phosphorus and available potassium, the N3 treatment showed the highest increases, while the S1 treatment had the smallest increase in available phosphorus, and the H1 treatment had the smallest increase in available potassium.

In the 20-40 cm soil layer, the treatments also exhibited similar trends regarding the effects on organic matter, total nitrogen, hydrolyzable nitrogen, available phosphorus, and available potassium. The J3 treatment resulted in the greatest increase in organic matter content, while the N1 treatment had the smallest increase, which did not reach significance. The T3 treatment had the largest increase in total nitrogen, while the J1 treatment showed the smallest increase. For hydrolyzable nitrogen, the T1 treatment had the smallest increase, whereas the J3 treatment had the largest. In terms of available phosphorus and available potassium, the H1 treatment had the smallest increase in available phosphorus, the S1 treatment had the smallest increase in available potassium, and the N3 treatment showed the highest increases for both indicators.

Additionally, following the application of organic materials, the pH, electrical conductivity, and water-soluble salt content of the 0-40 cm soil exhibited different trends. The N, J, S, and H groups significantly lowered soil pH, electrical conductivity, and water-soluble salt content, whereas the T group increased these indicators, with the T3 treatment showing the most significant changes.

#### 3.1.3 Effects of different treatments on plant agronomic traits and yield

From (**Table 3**), it can be seen that the effect of different organic material treatments on cotton plant height follows the trend: N group > T group > S group > J group > H group > CK. Among them, the N3 treatment had the most significant effect on plant height and stem diameter, increasing by 20.63% and 35.1%, respectively. Compared to the CK treatment, the N3 treatment showed the greatest improvement in fresh and dry weights, with above-ground fresh and dry weights increasing by 33.86% and 37.59%, respectively, and below-ground fresh and dry weights increasing by 13.59% and 22.23%. Additionally, the N3 treatment also achieved the highest increases in single boll weight and cotton yield, reaching significant levels compared to other treatment groups.

**Table 3:** Effects of different organic materials on agronomic characters and yield of cotton.

Treatment		Height(cm)	Stem Thick(mm)	Fresh Weight above Ground(g)	Underground fresh weight(g)	Dry weight above ground(g)	Dry weight of underground part(g)	Single Bell weight(g)	Output(kg)
N	N1	75.54± 4.78bc	9.6± 0.12c	76.45± 5.15bc	13.875± 0.81ab	36.721± 2.21c	5.698± 0.402cd	6.4124± 1.25b	431.13± 10.45c
	N2	81.22± 4.79b	9.81± 0.16c	83.76± 5.16b	14.008± 0.73a	41.236± 3.24ab	7.003± 0.355a	6.4897± 1.28b	438.93± 10.28b
	N3	89.66± 3.85a	11.2± 0.22a	92.16± 5.27a	14.578± 0.93a	44.878± 3.26a	7.156± 0.311a	6.5521± 1.26a	445.17± 12.44a
T	T1	73.55± 4.35c	9.7± 0.14hc	75.15± 4.15bc	12.972± 0.99b	35.647± 3.19c	5.974± 0.44c	6.0987± 1.31d	422.22± 9.25e
	T2	82.11± 3.44b	9.9± 0.12c	83.92± 4.28b	13.004± 0.81b	40.714± 2.15b	6.213± 0.313bc	6.1152± 1.29d	425.17± 9.55d
	T3	88.49± 3.52a	10.5± 0.29b	90.89± 4.27a	14.185± 0.74a	42.159± 3.16ab	6.634± 0.411b	6.2731± 1.25c	435.97± 10.04b
J	J1	72.15± 4.41c	9.22± 0.17d	75.15± 5.13bc	12.824± 0.88bc	32.105± 4.21d	5.096± 0.405d	6.1423± 1.28d	420.14± 9.88e
	J2	77.88± 4.34bc	10.11± 0.18bc	78.98± 5.15bc	13.2470.97 ± b	34.109± 3.23cd	5.197± 0.313d	6.2116± 1.24c	428.97± 10.01c
	J3	86.89± 3.36a	10.19± 0.26bc	88.29± 4.29ab	13.995± 0.86a	35.708± 2.29c	5.753± 0.407cd	6.2145± 1.31c	440.17± 11.27a
S	S1	69.8± 3.06d	9.7± 0.15c	72.17± 4.14c	12.578± 0.74c	34.189± 4.19cd	5.933± 0.258c	6.0932± 1.34d	423.69± 9.36d
	S2	76.9± 3.25bc	9.85± 0.19c	78.77± 4.27bc	12.989± 0.90b	36.751± 3.16c	6.023± 0.205c	6.2301± 1.24c	432.11± 10.08c
	S3	84.6± 4.28ab	10.5± 0.22b	86.48± 5.21ab	13.887± 0.83ab	39.716± 4.26b	6.412± 0.212b	6.2477± 1.26c	438.19± 10.11b
H	H1	68.6± 4.06d	9.06± 0.13de	69.97± 5.18d	12.851± 0.99bc	28.112± 2.22e	4.511± 0.411e	5.8997± 1.29e	420.66± 9.69e
	H2	74.5± 3.23d	9.15± 0.17d	76.18± 5.13bc	13.013± 0.79b	33.098± 3.21d	4.687± 0.509e	5.9714± 1.05e	426.73± 9.83d
	H3	83.6± 4.21ab	9.68± 0.16c	85.55± 4.17ab	13.848± 0.85ab	36.795± 3.29c	5.899± 0.313c	6.0231± 1.09d	430.18± 9.94c
CK	CK	67.0± 3.05e	8.12± 0.11e	67.98± 4.07e	11.927± 0.85d	29.112± 2.18e	4.547± 0.404e	5.8979± 1.07e	420.11± 8.55e

N, farm fertilizer; T, biochar; J, biological bacterial fertilizer; S, commercial organic fertilizer; H, mineral source potassium fulvic acid. 1:50% recommended amount, 2:100% recommended amount, 3:150% recommended amount.

Different lowercase letters indicate significant differences at the 0.05 probability level (P < 0.05), determined by one-way analysis of variance (ANOVA) and Duncan's post hoc test for significance.

#### 3.1.4 Impact of different treatments on soil microbial population

**Figure 3:**
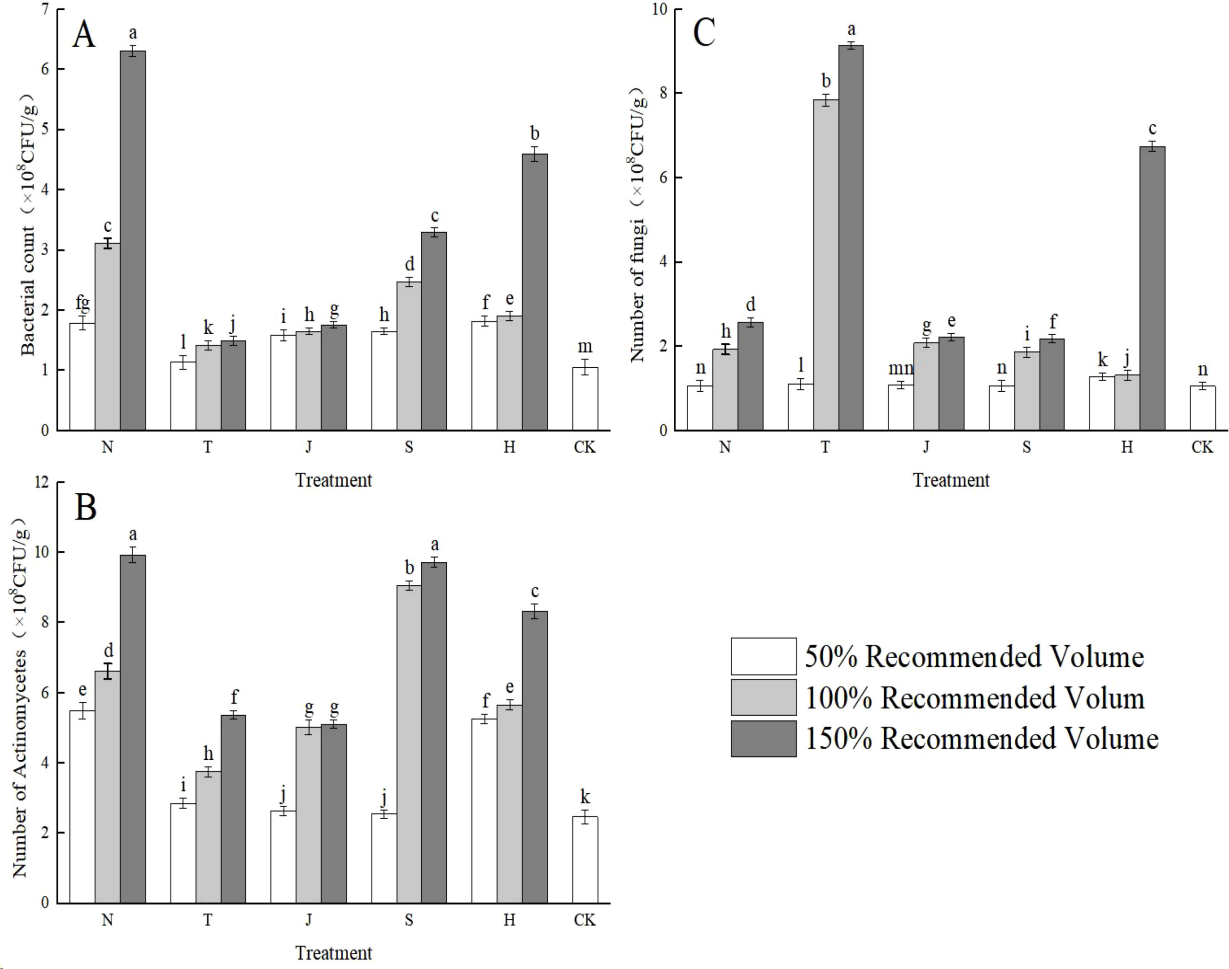
Effects of different organic materials on soil microbial population. Different lowercase letters indicate significant differences at the 0.05 probability level (P < 0.05), determined by one-way analysis of variance (ANOVA) and Duncan’s *post hoc* test for significance. The vertical bar chart represents the mean ± standard deviation (SD) calculated from three repetitions. N: farm fertilizer, T: biochar, J: biological bacterial fertilizer, S: commercial organic fertilizer, H: mineral source potassium fulvic acid. **(A)** number of bacteria, **(B)** number of fungi, **(C)** number of actinomycetes.

From (**Figure 3**), it is evident that the application of the five types of organic materials significantly increased the numbers of bacteria and actinomycetes in the soil compared to the control (CK) treatment. Except for the N1, J1, and S1 treatments, which did not show significant increases in fungal numbers, the other treatments exhibited notable effects on enhancing fungal populations. As shown in (**Figure 3A**), the N group had the most significant effect on bacterial counts, with increases ranging from 70.16% to 500.95%, with the N3 treatment achieving the highest bacterial numbers. (**Figure 3B**) indicates that the T group had the greatest increase in fungal numbers, ranging from 4.41% to 765.27%, with the T3 treatment reaching the peak fungal count. According to (**Figure 3C**), the N group also showed the most substantial increase in actinomycete counts, with a range of 123.14% to 302.85%, again with the N3 treatment yielding the highest actinomycete numbers.

### 3.2 Comprehensive evaluation

**Figure 4:**
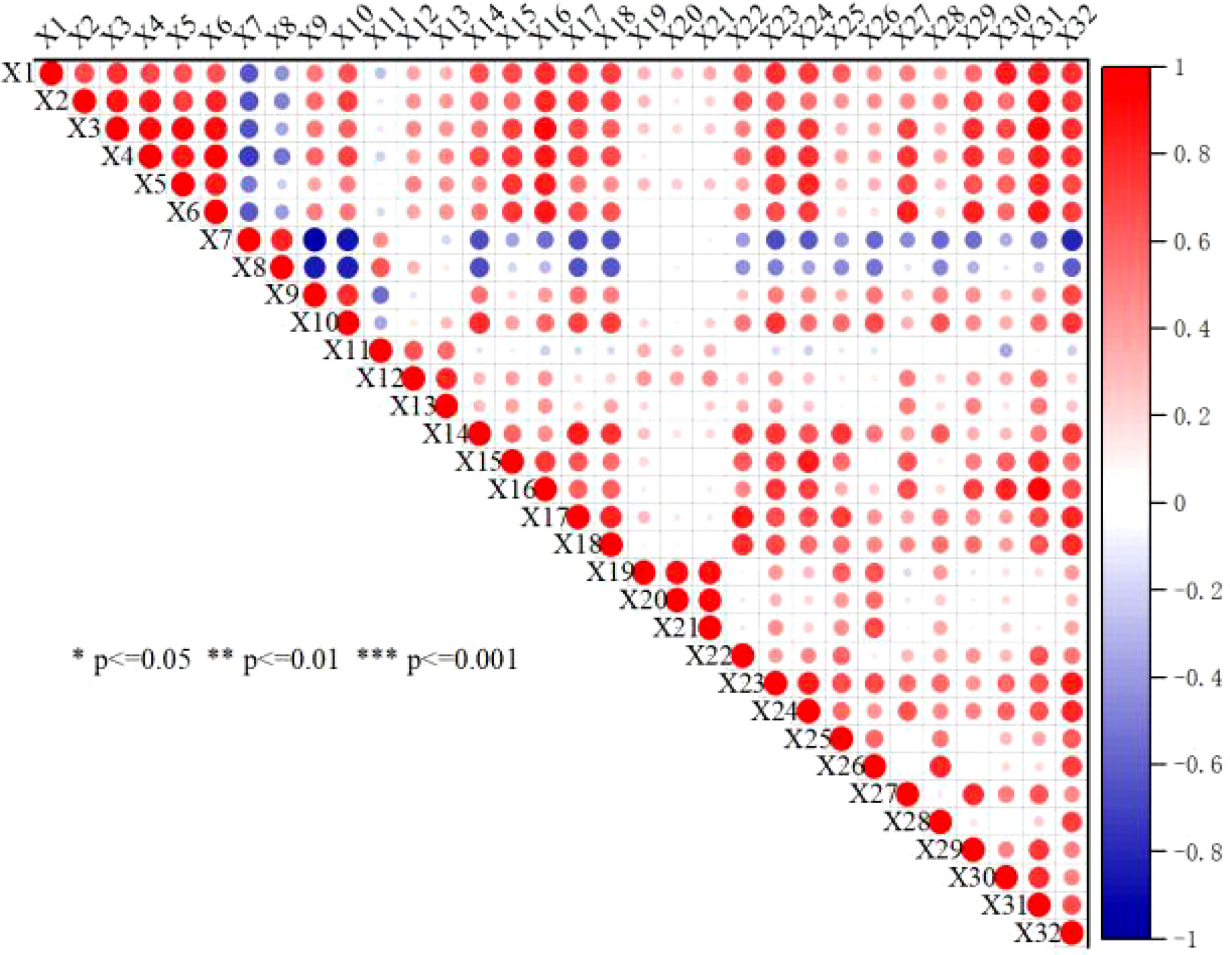
Correlation analysis between soil index and plant index.

**Figure 5:**
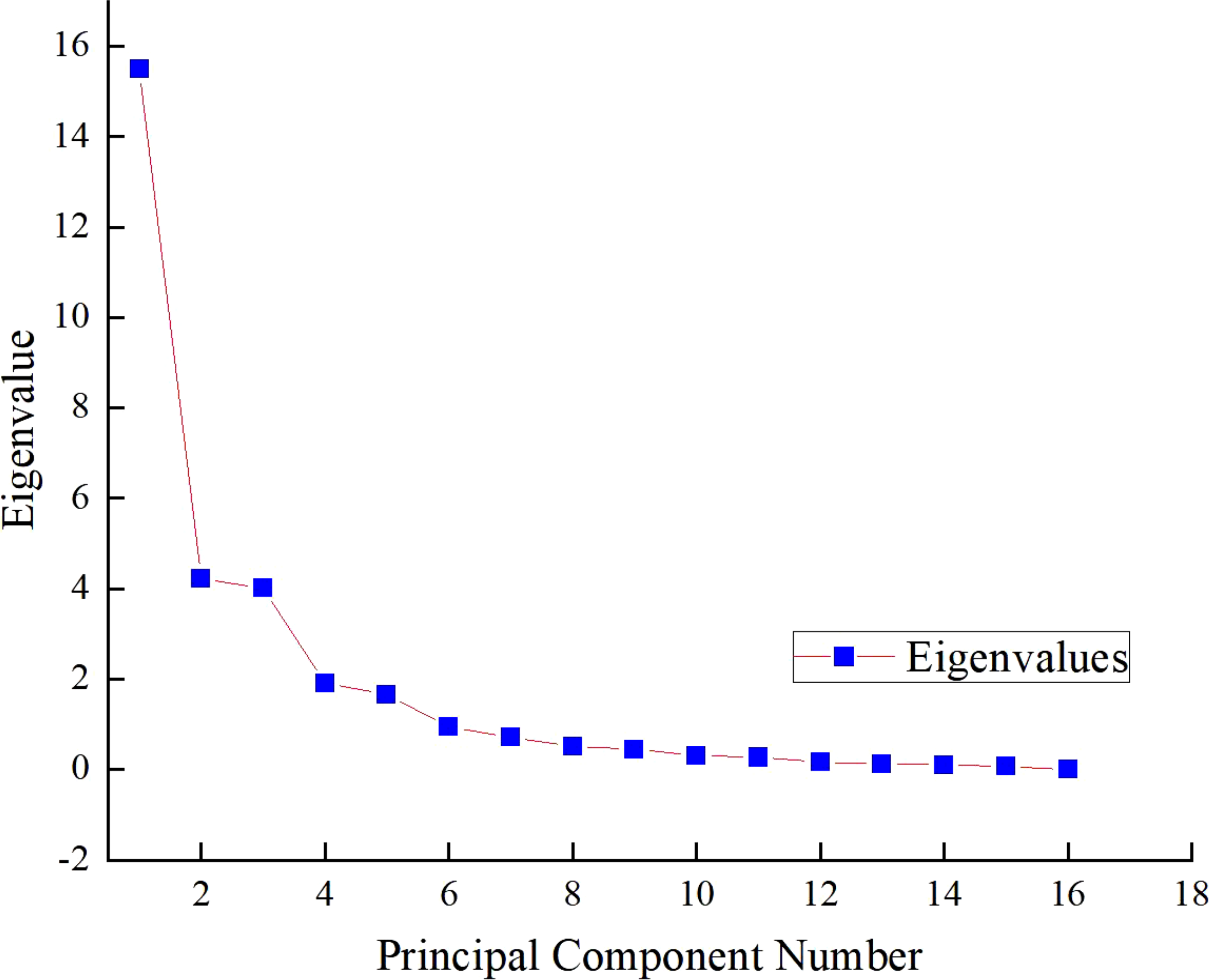
Rubble maps.

**Figure 6:**
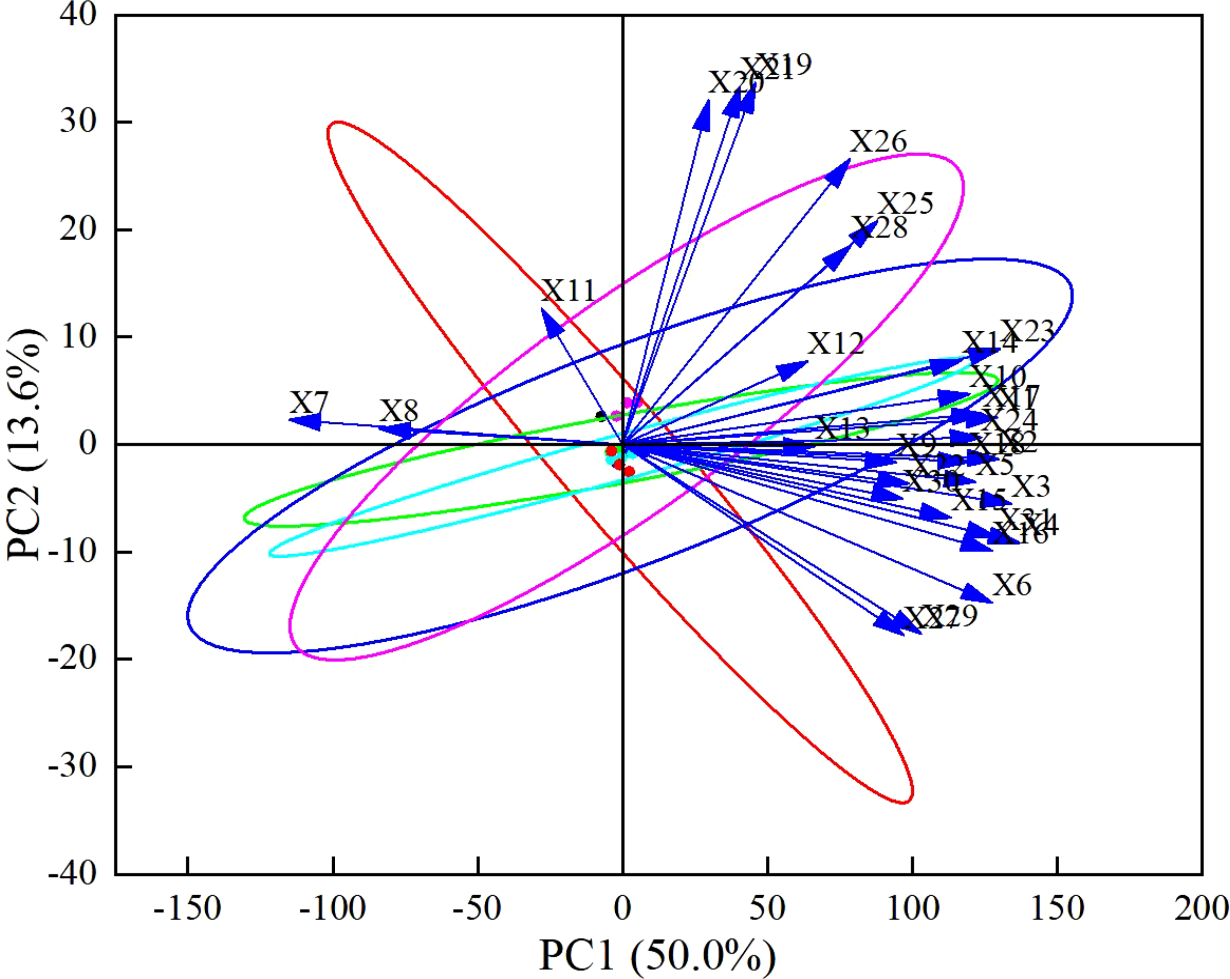
Principal component analysis.X1: Plant height, X2: Stem diameter, X3: Aboveground fresh weight, X4: Belowground fresh weight, X5: Aboveground dry weight, X6: Belowground dry weight, X7: Bulk density 0-20cm, X8: Bulk density 20-40cm, X9: Porosity 0-20cm, X10: Porosity 20-40cm, X11: pH 0-20cm, X12: Soluble salts 0-20cm, X13: Electrical conductivity 0-20cm, X14: Hydrolyzable nitrogen 0-20cm, X15: Available phosphorus 0-20cm, X16: Quick-acting potassium 0-20cm, X17: Organic matter 0-20cm, X18: Total nitrogen 0-20cm, X19: pH 20-40cm, X20: Soluble salts 20-40cm, X21: Electrical conductivity 20-40cm, X22: Hydrolyzable nitrogen 20-40cm, X23: Available phosphorus 20-40cm, X24: Quick-acting potassium 20-40cm, X25: Organic matter 20-40cm, X26: Total nitrogen 20-40cm, X27: Bacteria 0-40cm, X28: Fungi 0-40cm, X29: Actinomycetes 0-40cm, X30: Single ring weight, X31: Yield, X32: Organic matter content in organic materials.

This study conducted a correlation analysis on 31 indicators related to soil, cotton seedlings, and yield across different treatments (**Figure 4**). The results indicated a certain degree of discrete correlation among the indicators, albeit with varying levels of correlation. To further explore the relationships between physiological indicators, biplots of principal components were generated (**Figures 5**, **6**), showing a connection between indicator X1 (plant height) and X3 (fresh weight of aerial parts). This information reflects the differences in how various indicators influence the alleviation of soil compaction.

Using SPSS 26.0, a principal component analysis was performed on the 31 indicators, yielding a Kaiser-Meyer-Olkin (KMO) value of 0.789, indicating the data’s suitability for this analysis. The cumulative contribution rate of the first three principal components was 76.619%, with specific contributions of 50.046% for the first component (PC1), 13.626% for the second (PC2), and 12.948% for the third (PC3). All eigenvalues were greater than 1, aligning with selection criteria, thus these three components were chosen as the main evaluation factors for soil improvement.

The analysis of eigenvectors revealed that PC1 primarily included indicators such as plant height, stem diameter, fresh weight of aerial parts, and fresh weight of underground parts, with significant contributions from the eigenvectors: 0.22179 (plant height), 0.22266 (stem diameter), 0.23039 (fresh weight of aerial parts), and 0.23498 (fresh weight of underground parts). PC2 was mainly associated with pH, soluble salts, and electrical conductivity at the 20-40 cm depth, while PC3 primarily involved soil bulk density and microbial counts.

#### 3.2.1 Correlation analysis between soil index and plant index

The correlation analysis (**Figure 4**) further revealed significant relationships between organic matter content and various soil indicators. Specifically, it was significantly negatively correlated with bulk density at 20-40 cm (p < 0.05) and extremely negatively correlated with bulk density at 0-20 cm (p < 0.001). Additionally, it showed significant positive correlations with available phosphorus at 0-20 cm and hydrolyzable nitrogen at 20-40 cm (p < 0.05). There were also significant positive correlations with dry weights of both aerial and underground parts, as well as with soil porosity at 0-20 cm and microbial counts (p < 0.01). These results suggest that the application of organic materials can improve soil structure, reduce bulk density, promote microbial proliferation, and enhance nutrient utilization efficiency.

#### 3.2.2 Cluster analysis between soil index and plant index

**Figure 7:**
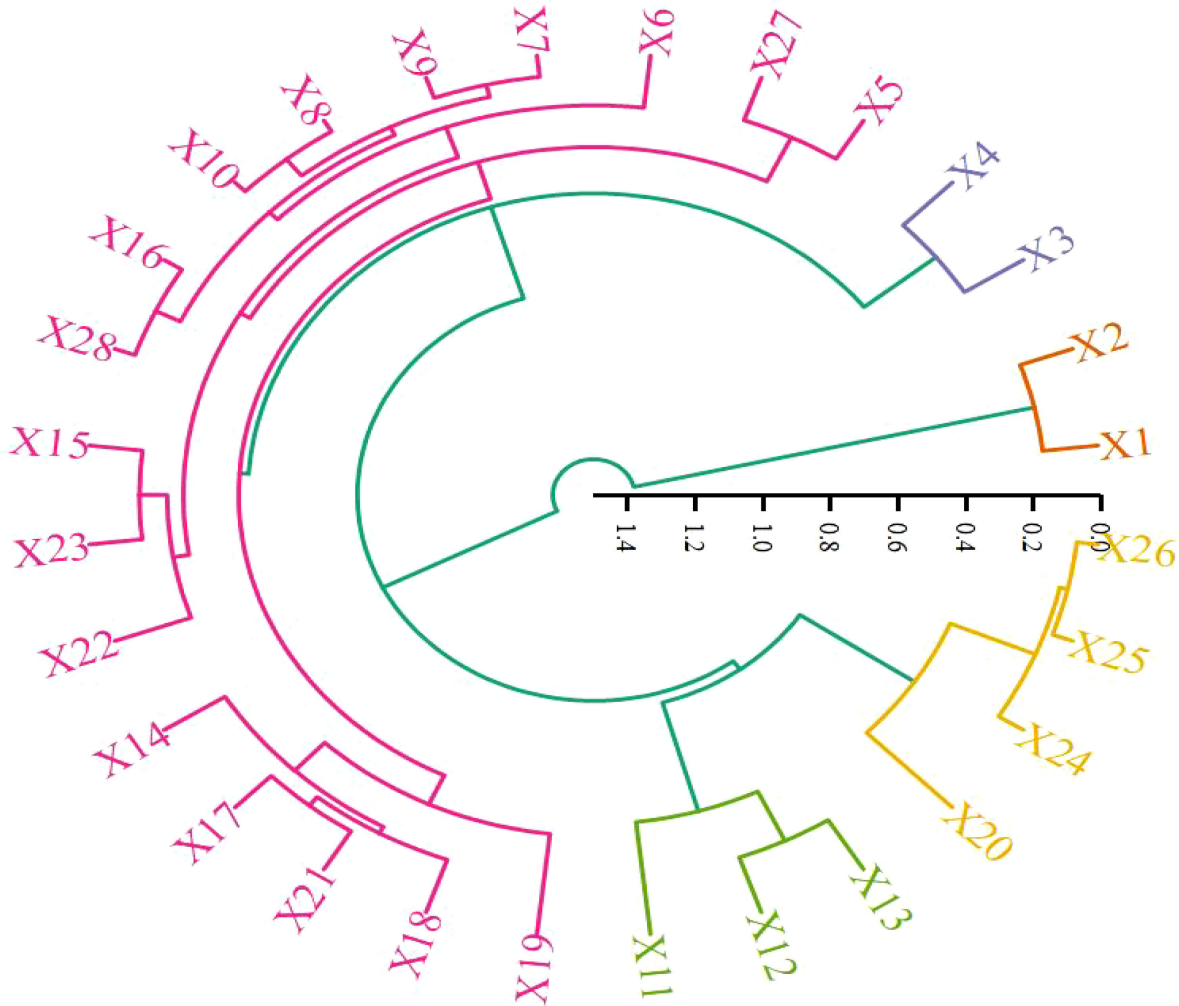
Cluster analysis between soil index and plant index.X1: Plant height, X2: Stem diameter, X3: Aboveground fresh weight, X4: Belowground fresh weight, X5: Aboveground dry weight, X6: Belowground dry weight, X7: Bulk density 0-20cm, X8: Bulk density 20-40cm, X9: Porosity 0-20cm, X10: Porosity 20-40cm, X11: pH 0-20cm, X12: Soluble salts 0-20cm, X13: Electrical conductivity 0-20cm, X14: Hydrolyzable nitrogen 0-20cm, X15: Available phosphorus 0-20cm, X16: Quick-acting potassium 0-20cm, X17: Organic matter 0-20cm, X18: Total nitrogen 0-20cm, X19: pH 20-40cm, X20: Soluble salts 20-40cm, X21: Electrical conductivity 20-40cm, X22: Hydrolyzable nitrogen 20-40cm, X23: Available phosphorus 20-40cm, X24: Quick-acting potassium 20-40cm, X25: Organic matter 20-40cm, X26: Total nitrogen 20-40cm, X27: Bacteria 0-40cm, X28: Fungi 0-40cm, X29: Actinomycetes 0-40cm, X30: Single ring weight, X31: Yield, X32: Organic matter content in organic materials.

Cluster analysis (**Figure 7**) categorized the indicators from various treatments into five groups, with representative indicators including plant height, fresh weight of aerial parts, bulk density at 20-40 cm, soluble salts at 0-20 cm, and total nitrogen at 20-40 cm. This indicates that the application of organic materials not only promotes cotton growth but also effectively alleviates soil compaction.

##### 3.2.2.1 The first principal component

F1 = 0.22X1 + 0.22X2 + 0.23X3 + 0.23X4 + 0.21X5 + 0.22X6+-0.20X7-0.14X8 + 0.16X9 + 0.21X10-0.05X11 + 0.11X12 + 0.11X13 + 0.20X14 + 0.20X15 + 0.22X16 + 0.21X17 + 0.21X18 + 0.08X19 + 0.05X20 + 0.07X21 + 0.17X22 + 0.22X23 + 0.22X24 + 0.15X25 + 0.13X26 + 0.17X27 + 0.14X28 + 0.18X29 + 0.17X30 + 0.22X31.

##### 3.2.2.2 The second principal component

F2 = 0.03X1-0.02X2-0.07X3-0.11X4-0.04X5-0.19X6 + 0.03X7 + 0.02X8-0.02X9 + 0.06X10 + 0.16X11 + 0.10X12-0.01X13 + 0.10X14-0.09X15-0.12X16 + 0.04X17-0.02X18 + 0.42X19 + 0.40X20 + 0.42X21-0.05X22 + 0.11X23 + 0.01X24 + 0.26X25 + 0.33X26-0.22X27 + 0.23X28-0.22X29-0.06X30-0.11X31.

##### 3.2.2.3 The third principal component

F3 = 0.02X1 + 0.02X2 + 0.11X3 + 0.02X4 + 0.16X5 + 0.08X6 + 0.24X7 + 0.39X8-0.29X9-0.21X10 + 0.36X11 + 0.38X12 + 0.29X13-0.13X14 + 0.13X15 + 0.11X16-0.11X17-0.10X18 + 0.14X19 + 0.14X20 + 0.14X21-0.04X22-0.01X23 + 0.02X24-0.11X25-0.16X26 + 0.18X27-0.15X28 + 0.11X29 + 0.12X30 + 0.15X31.

Based on the variance contribution analysis, the first three principal components explain 50.05%, 13.63%, and 12.95% (**Table 4**) of the variance, respectively. Combining the principal component coefficients and their variance contributions, the comprehensive evaluation formula is: F=50.05F1 + 13.63F2 + 12.95F3 (**Table 5**). Using this formula, the comprehensive scores for the five types of organic materials and their different application rates on alleviating soil compaction, cotton seedling growth, and yield were calculated (**Table 6**). The results indicate that the effectiveness in alleviating soil compaction is ranked as follows:N3>T3>S3>J3>N2>T2>H3>J2>S2>T1>N1>H2>CK. Specifically, the 150% and 100% recommended rates of farmyard manure, biochar, microbial fertilizer, commercial organic fertilizer, and potassium humate from mineral sources, as well as the 50% recommended rates of farmyard manure and biochar, showed significant effects in alleviating soil compaction, while the other treatments did not demonstrate significant effects.

**Table 4:** Principal component analysis eigenvalue and contribution rate.

Ingredients	Initial eigenvalue			Extract the sum of squares of loads		
	Total	Percentage of variance	Cumulative (%)	Total	Percentage of variance	Cumulative (%)
1	15.51	50.05	50.05	15.51	50.05	50.05
2	4.22	13.63	63.67	4.22	13.63	63.67
3	4.01	12.94	76.61	4.01	12.95	76.62

**Table 5:** Principal component index load matrix and eigenvector.

Variable	Eigenvectors and eigenvalues			Load matrix		
	Principal component 1	Principal component 2	Principal component 3	Principal component 1	Principal component 2	Principal component 3
X1	0.22	0.03	0.02	0.87	0.06	0.03
X2	0.22	-0.02	0.02	0.87	-0.03	0.04
X3	0.23	-0.07	0.11	0.90	-0.14	0.22
X4	0.23	-0.11	0.02	0.92	-0.23	0.04
X5	0.21	-0.04	0.16	0.82	-0.09	0.31
X6	0.22	-0.18	0.08	0.86	-0.37	0.15
X7	-0.20	0.03	0.24	-0.77	0.05	0.47
X8	-0.14	0.02	0.39	-0.56	0.03	0.78
X9	0.16	-0.02	-0.29	0.63	-0.04	-0.57
X10	0.21	0.06	-0.21	0.80	0.12	-0.41
X11	-0.05	0.16	0.36	-0.18	0.32	0.71
X12	0.11	0.10	0.38	0.43	0.19	0.76
X13	0.11	-0.01	0.29	0.44	-0.01	0.57
X14	0.20	0.10	-0.13	0.79	0.20	-0.26
X15	0.19	-0.09	0.13	0.76	-0.17	0.25
X16	0.22	-0.12	0.11	0.86	-0.25	0.22
X17	0.21	0.04	-0.11	0.84	0.07	-0.22
X18	0.21	-0.02	-0.11	0.80	-0.04	-0.21
X19	0.08	0.42	0.14	0.30	0.86	0.27
X20	0.05	0.40	0.14	0.20	0.82	0.27
X21	0.07	0.42	0.14	0.27	0.85	0.27
X22	0.17	-0.05	-0.04	0.66	-0.09	-0.07
X23	0.22	0.11	-0.01	0.88	0.23	-0.00
X24	0.21	0.01	0.02	0.83	0.02	0.04
X25	0.15	0.26	-0.11	0.59	0.53	-0.22
X26	0.13	0.33	-0.16	0.53	0.68	-0.31
X27	0.17	-0.22	0.18	0.65	-0.45	0.35
X28	0.14	0.23	-0.15	0.53	0.47	-0.29
X29	0.18	-0.22	0.11	0.69	-0.45	0.21
X30	0.17	-0.06	0.11	0.65	-0.13	0.23
X31	0.22	-0.11	0.15	0.87	-0.22	0.29

**Table 6:** Principal component score table.

Treatment	F1	F2	F3	F synthesis	Rank
CK	-1.87	1.26	2.65	-42.22	13
N1	-0.49	-0.83	0.25	-32.74	11
N2	0.61	-0.35	0.44	31.71	5
N3	1.88	-0.38	1.89	113.67	1
T1	-0.53	1.29	-0.79	-19.36	10
T2	0.37	1.90	-1.19	28.68	6
T3	1.28	1.90	-0.46	83.69	2
J1	-1.06	-0.34	-0.58	-65.22	14
J2	-0.28	-0.18	-0.04	-17.19	8
J3	0.95	-0.14	-0.18	43.18	4
S1	-1.00	-0.68	-0.59	-67.13	16
S2	-0.09	-0.61	-0.35	-17.45	9
S3	0.89	-0.41	0.50	45.35	3
H1	-0.96	-0.30	-0.51	-58.54	15
H2	-0.29	-0.91	-0.53	-33.57	12
H3	0.61	-1.23	-0.50	7.18	7

N, farm fertilizer; T, biochar; J, biological bacterial fertilizer; S, commercial organic fertilizer; H, mineral source potassium fulvic acid. 1:50% recommended amount, 2:100% recommended amount, 3:150% recommended amount.

### 3.3 Structural equation model

**Figure 8:**
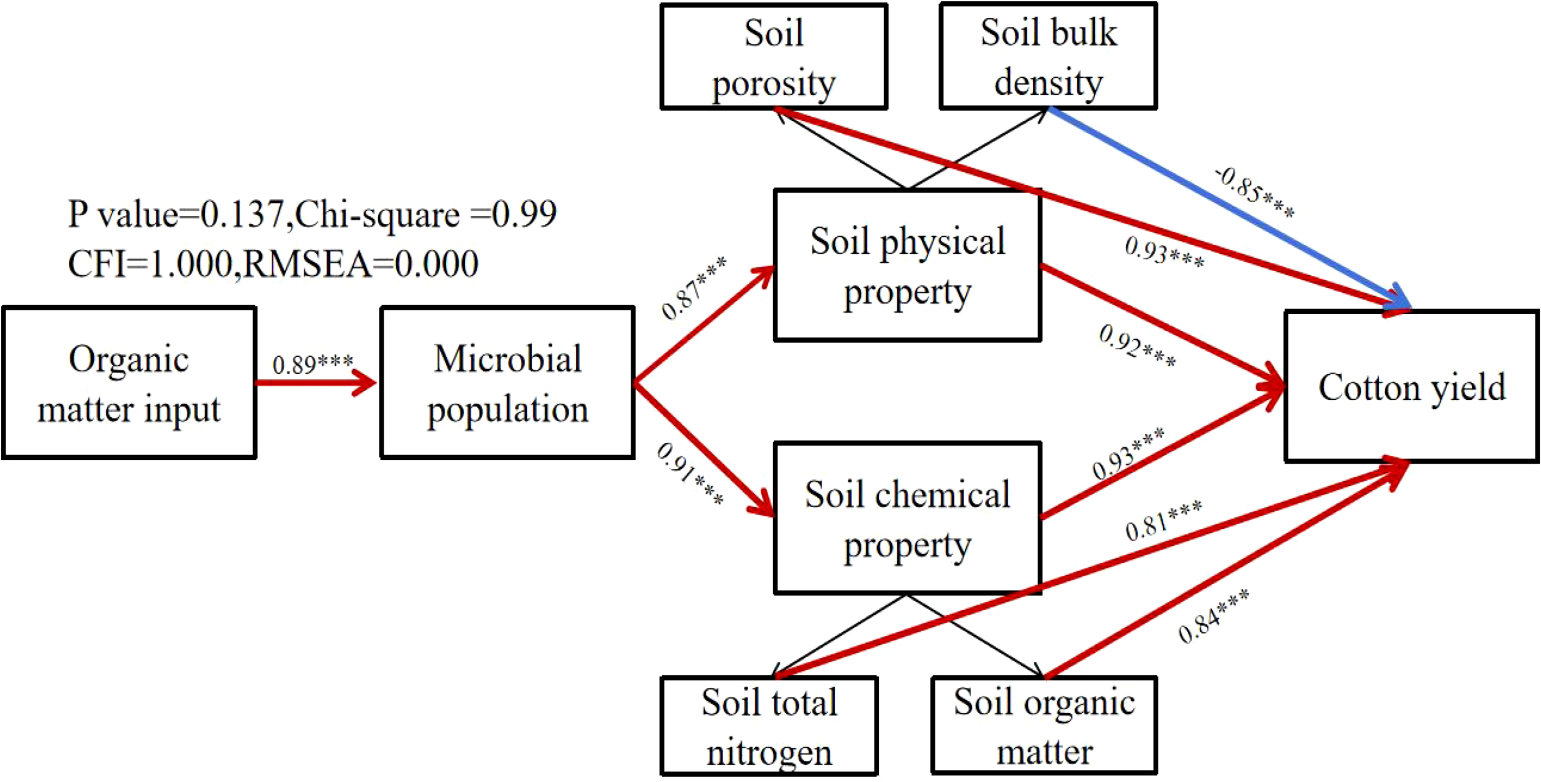
Structural equation model diagram. ***p < 0.001.

We used structural equation model (SEM) to investigate the effects of organic matter input on soil microbial quantity, soil physicochemical properties and cotton yield (P value=0.138,Chi-square =0.99, CFI=1.000,RMSEA=0.000)(**Figure 8**). The results showed that the number of microorganisms was strongly responsive to the input of organic matter, and the effect of organic matter on soil chemical properties was the largest. Soil physical properties and soil chemical properties have a strong response to the number of microorganisms, and the effect value of the number of microorganisms on the interaction path of soil chemical properties is the largest. Soil physical properties and soil chemical properties have a strong response to the yield, and soil bulk density in soil physical properties has a negative response to the yield, which also indicates that the smaller the soil bulk density, the higher the cotton yield, which is consistent with the experimental results of this study. The mechanism further confirmed by this model is as follows: the input of organic matter mainly regulates the number of microorganisms and the richness of microbial species, and then improves the physicochemical properties of soil, thereby increasing the cotton yield.

## 4 Discussion

### 4.1 Effects of different organic materials on soil physical properties

In this study, the application of organic materials significantly increased the sand content in cotton fields while reducing the content of clay and silt particles. Soil bulk density and porosity are important indicators for assessing soil structure. High bulk density indicates increased soil density and reduced aggregate structure, while low bulk density reflects higher porosity and better soil structure (Yao et al., 2019). demonstrated that the application of organic materials can reduce soil bulk density, increase the content of organic matter, nitrogen, phosphorus, and potassium, and enhance the number of beneficial microorganisms, ultimately improving soil enzyme activity and promoting crop yield. Zhang et al. further supported this finding, stating that organic fertilizers positively impact crop growth and soil improvement (Zhao et al., 2025). In our experiment, all five organic materials significantly reduced soil bulk density to varying degrees, with biochar showing the most pronounced effect. The advantages of biochar in improving soil structure and water retention capacity have been well established. Its unique porous structure interacts with soil aggregates, increasing overall porosity and altering pore size distribution (Oguntunde et al., 2008; Abel et al., 2013; Petersen et al., 2016; Werdin et al., 2020). Studies have shown that compared to the control group, the application of biochar significantly improved soil overall porosity and saturated hydraulic conductivity while reducing soil bulk density. Additionally, as the application rate increased, both the bulk density and specific gravity of the 0-40 cm soil layer significantly decreased (Oguntunde et al., 2008). In this experiment, the T2 treatment (biochar application) significantly reduced soil bulk density, while the other four organic materials also contributed to this reduction to varying extents. Among them, T2 and T3 treatments showed the most significant improvement in soil porosity at depths of 0-20 cm and 20-40 cm. This may be attributed to the role of humic substances in organic materials as key binding agents that promote the formation of good soil structure. This process enhances the soil’s thermal absorption capacity, improves fertility, reduces soil compaction, and consequently increases porosity. These changes facilitate the rapid exchange of water, soil, and air, ultimately leading to a decrease in soil bulk density (Zheng et al., 2012; Seiji and Nobuhisa, 2013; Mi et al., 2018; Ju et al., 2022).

### 4.2 Effects of different organic materials on soil chemical properties and salinity

The application of organic materials significantly impacts soil properties and structure, altering nutrient transformation processes, reducing nutrient loss, and enhancing crop nutrient absorption and utilization, thereby promoting plant growth (Study on the effect of biomass modifier on sandy soil, 2023). Organic materials are rich in organic matter, nitrogen, phosphorus, potassium, and other mineral elements, which not only replenish nutrients lost due to mineralization and decomposition but also improve the availability of minerals like potassium and phosphorus (Liu et al., 2023).Jiang et al. indicated that the application of amendments could increase organic matter, total nitrogen, and available nutrients in the soil (Jiang et al., 2011). Furthermore, Song et al. found that applying cattle manure significantly increased the levels of available phosphorus, available potassium, total nitrogen, and alkaline nitrogen in the soil (Song et al., 2017). Our study results show that the application of organic fertilizers led to significant increases in total nitrogen, available phosphorus, available potassium, and organic matter content in the soil. These increases in available nutrients have a positive effect on crop biological absorption and utilization. Moreover, the overall performance of the N treatment group was superior to that of other treatment groups, possibly due to the higher microbial and enzyme content in cattle manure, which facilitated the mineralization of available nutrients and accelerated their accumulation in the soil. The potassium humic acid from mineral sources contains humic acid, fulvic acid, and other organic macromolecules, which react with alkaline substances in the soil upon application, leading to a decrease in soil pH. However, the impact of farm manure and biochar on soil pH was minimal, likely due to their strong acid-base buffering capacity. It is noteworthy that as the application of biochar increased, the soil pH exhibited an upward trend, which may be related to the inherent alkalinity of biochar. The functional groups in biochar (such as ester and ether bonds) and the cations released (such as potassium, calcium, and magnesium) collectively contributed to the increase in soil pH (Mi et al., 2018).

### 4.3 Effects of different organic materials on agronomic traits, yield and microorganisms of cotton

The damage to clay soil structure can lead to soil hardening and decreased oxygen availability, severely hindering the growth of crop roots. Reduced root vitality and diminished cellular respiration lead to insufficient energy levels, consequently affecting the roots’ ability to absorb nutrients from the soil. This nutrient deficiency negatively impacts the growth of the above-ground plant parts, ultimately resulting in decreased yield and quality (Wang, 2018).Research indicates that the application of organic materials can significantly enhance the yield and quality of various crops, including tobacco, ginger, cassava, and cucumber (Bending et al., 2002; Zhang et al., 2018; Yao et al., 2019; Bai et al., 2020). By improving nutrient utilization efficiency, organic materials promote the growth of cotton seedlings, reflected in increased plant height and stem diameter (Sun et al., 2019). The application of five different organic fertilizers at varying doses significantly improved the cotton’s plant height, stem diameter, dry weight, fresh weight, boll weight, and overall yield. Among them, the N3 treatment had the most pronounced impact on cotton agronomic traits, attributed to several factors: 1. Rich microbial content: Farm manure is abundant in beneficial microorganisms that can fix nitrogen and enhance the availability of phosphorus and potassium in the soil; 2. Symbiotic relationships: These beneficial microorganisms form symbiotic relationships with plant roots, optimizing the rhizosphere environment and stimulating plant growth (Zhang et al., 2010); 3. Plant growth hormones: Microorganisms can produce plant growth hormones and engage in biological control, thereby reducing the impact of pathogenic infections (Rouzi et al., 2011; Zhao et al., 2012).The application of organic materials introduces rich organic matter into the soil, establishing new biological systems and providing microorganisms with abundant nutrients and energy. This significantly enhances microbial activity and reproductive capacity (Ortega et al., 2016). The physiological activities of microorganisms not only decompose organic matter into nutrients that can be absorbed by crops but also synthesize new organic compounds that promote the continuous accumulation of soil nutrients. Additionally, microorganisms can release nutrients fixed in the soil and absorb those that are prone to loss, thereby enhancing the nutrient supply and storage capacity of the soil (Liu et al., 2022). The application of organic and biological fertilizers helps to enrich soil nutrients, increase microbial biomass, and optimize microbial community structure (Tan et al., 2007; Wang et al., 2018).In this study, the application of different types and amounts of organic materials significantly affected the microbial population in the soil. The application of organic materials provided rich carbon and nitrogen sources for the soil, leading to a significant increase in the number of bacteria, fungi, and actinomycetes (Gryta et al., 2020). Li et al (Li et al., 2019). found that both the individual application of organic fertilizers and their combined application with inorganic fertilizers significantly increased the populations of bacteria, fungi, and nitrogen-fixing bacteria in the soil compared to the control group. Among the treatments, the N group exhibited the most pronounced microbial population. This effect may be attributed to the high nutrient demand of crops during their growth stages, accelerating the decomposition of soil organic matter. Furthermore, the nutrients released during microbial decomposition support the growth and reproduction of these organisms, further enhancing their reproductive and metabolic activities. This process improves soil enzyme activity and enhances the soil’s ability to retain water and nutrients. The competitive dynamics among microbial populations may also facilitate the dominance of beneficial microorganisms in farm manure. Previous studies have shown that the application of organic materials significantly increases the number of bacteria in the rhizosphere soil of crops, while the number of fungi decreases. This change indicates an enhancement in the richness and functional diversity of microbial communities, accompanied by an increase in enzyme activities (such as sucrase and urease) (Bending et al., 2002; Zhang et al., 2013). Therefore, farm fertilizers demonstrate superior effects on improving soil physical and chemical properties compared to general organic materials. Particularly, the microbial fermentation of cattle manure (bio-organic fertilizer) provides specific benefits for soil protection and yield enhancement, aligning closely with the goals of sustainable agricultural development and thus warranting further promotion. The aim of this study is to screen organic materials with different functions for soil improvement and to provide data support for subsequent experiments. We will continue to focus on the impact of applying organic materials on crop yield and growth under saline-alkali soil conditions, and explore the optimal application strategies for different organic materials.

## 5 Conclusions

This study showed that among the five organic materials, farm manure was an effective way to alleviate soil compaction and promote cotton growth. By adding farm manure to clay, soil bulk density and salinity were significantly reduced, and soil organic matter, total nitrogen and available nutrients were increased, thus promoting the growth of cotton under clay stress. The optimal addition amount is 36,000 Kg HM-2, which provides a new idea for the effective use of farm fertilizer and the improvement of bonding clay. At the same time, the application of farm manure is in line with sustainable development requirements, contributes to soil health and crop productivity, and is worth promoting in broader agricultural practices. In addition, future research could explore the effects of farm manure in different regions and soil types to further validate its important role in sustainable agriculture.

## Data Availability

The raw data supporting the conclusions of this article will be made available by the authors, without undue reservation.
